# Molecular diagnosis of Cytomegalovirus infection: clinical performance of the Aptima transcription-mediated amplification assay toward conventional qPCR chemistry on whole blood samples

**DOI:** 10.1128/jcm.00906-23

**Published:** 2024-02-13

**Authors:** Paolo Bottino, Lisa Pastrone, Elisa Zanotto, Francesca Sidoti, Rossana Cavallo, Cristina Costa

**Affiliations:** 1 S.C. Microbiology and Virology U, A.O.U. “Città della Salute e della Scienza di Torino”, Turin, Italy; Mayo Clinic Minnesota, Rochester, Minnesota, USA

**Keywords:** HCMV, transcription-mediated amplification, qPCR, Aptima, whole blood

## Abstract

Human Cytomegalovirus (HCMV) infection is life-threatening for immunocompromised patients. Quantitative molecular assays on whole blood or plasma are the gold standard for the diagnosis of invasive HCMV infection and for monitoring antiviral treatment in individuals at risk of HCMV disease. For these reasons, an accurate standardization toward the WHO 1st International Standard among different centers and diagnostic kits represents an effort for better clinical management of HCMV-positive patients. Herein, we evaluate, for the first time, the performance of a new transcription-mediated amplification (TMA) assay versus quantitative polymerase chain reaction (qPCR) chemistry, used as a routine method, on whole blood samples. A total of 755 clinical whole blood specimens were collected and tested simultaneously with TMA and qPCR assays. The data showed a qualitative agreement of 99.27% for positive quantified samples and 89.39% for those undetected between the two tested methods. Evaluation of viremia in positive samples highlighted a good correlation between TMA and qPCR chemistries in terms of International Units (ΔLog_10_ IU/mL: −0.29 ± 0.40). The TMA assay showed a significant correlation with qPCR in patients monitored for up to 3 months, thus allowing an accurate assessment of viremia in transplant patients. Therefore, TMA chemistry showed good agreement with qPCR testing, used as a current diagnostic routine. It also offers important advantages, such as FDA approval on plasma and *In Vitro* Diagnostic (IVD) on both plasma and whole blood, automated workflow with minimal hands-on time, and random access loading, thus enabling a rapid and reliable diagnostic in HCMV-infected patients.

**IMPORTANCE:**

In this paper, we describe the clinical performance of a novel transcription-mediated amplification (TMA) assay for the detection and quantification of human Cytomegalovirus (HCMV) DNA from whole blood samples. This is a pivotal analysis in immunocompromised patients [transplanted, HIV-positive, and Hematopoietic Stem Cell (HSC) recipients], and molecular tests with high sensitivity and specificity are necessary to evaluate the HCMV viral load in these patients. To our knowledge, this is the first in-depth evaluation of TMA chemistry for HCMV diagnosis on whole blood samples. Moreover, also technical aspects of this assay make it suitable for clinical diagnostics.

## INTRODUCTION

Human Cytomegalovirus (HCMV) is a ubiquitous herpesvirus that is highly widespread throughout the world with a seroprevalence rate among women of reproductive age ranging from 100% in Africa and Asia to 45.6%–95.7% in Europe and North America (1). Focusing on Italy, HCMV seroprevalence appears to reach 70%–80% (2, 3).

HCMV infection can occur throughout life, from pregnancy phase to adulthood, via several transmission routes. As congenital HCMV infection, primary and non-primary maternal infection (exogenous reinfection with a different strain or endogenous viral reactivation) of the virus during pregnancy can result in *in utero* transmission to the developing fetus (vertical route) (4, 5). In the postnatal period, HCMV is transmitted through exposure to different biological fluids (saliva, stool, and urine) as a consequence of community exposure or by genital secretions, especially in the adult stage (1, 3, 6, 7). Another important route of transmission of primary infection is solid organ transplantation, especially in cases where there is a serological mismatch between donor and recipient (HCMV-seronegative recipient/HCMV-seropositive donor) (8). Otherwise, infection may occur as reactivation in those patients with risk factors such as intense immunosuppression, acute rejection, advanced age of the donor and/or the recipient, other viral infections, and genetic polymorphisms (9, 10).

HCMV infection is usually asymptomatic in immunocompetent people, although clinical symptoms of primary infection may include a nonspecific glandular fever syndrome (mononucleosis) characterized by flu-like symptoms (11). However, in immunocompromised or transplant patients, HCMV primary infection or reactivation represents a fearsome complication resulting in a viral syndrome, characterized by fever and malaise as well as leukopenia, thrombocytopenia, and increased liver enzymes. Sometimes, severe complications (e.g., pneumonia, hepatitis, meningoencephalitis, myocarditis, and opportunistic infections) may occur, requiring admission to an intensive care unit (8, 12).

Direct detection of viral DNA by quantitative polymerase chain reaction (qPCR) is currently the standard of care (SoC) for the diagnosis of HCMV infection (13, 14), and several commercial kits based on conserved genetic regions have been developed to detect and quantify viral DNA from different biological matrices (15–19). Whole blood and plasma represent the most common specimens for the diagnosis (20–22), although cerebrospinal fluid and bronchoalveolar lavage are sometimes used (23). Scheduled monitoring of HCMV viremia in immunocompromised individuals is critical to identify patients at risk for HCMV disease, evaluate preemptive therapy, and assess response to treatment (14).

In addition to qPCR, other molecular techniques such as transcription-mediated amplification (TMA) have been developed and approved as commercial kits for the diagnosis of infectious diseases (24). Furthermore, TMA has been recognized as one of the most sensitive detection assays for the evaluation of HCV and HIV-1, with excellent performance compared to qPCR (25, 26). More recently, TMA has been extended to the diagnosis and monitoring of HCMV infection in transplant patients. However, its clinical performance, especially on whole blood, is still being studied.

Starting from this perspective, the main aim of our work was to evaluate the clinical performance of TMA chemistry on whole blood samples in a cohort of adult and pediatric individuals. Furthermore, focusing on transplant patients, HCMV viremia was monitored over time with both chemistries.

## MATERIALS AND METHODS

### Setting and study design

The University Hospital “Città della Salute e della Scienza di Torino” (Turin, Italy) is a regional tertiary hospital with 1,900 beds spread over three facilities: a general hospital, a pediatric clinic, and a rehabilitation department. It represents a center of excellence in solid organ (mainly kidney and liver) and tissue (corneas, skin, and skeletal muscle) transplantation for adult and pediatric patients.

The study was performed on 755 whole blood samples collected from 323 patients (273 adults and 50 children under 18 years of age) in the period May–November 2022 at the Microbiology and Virology Laboratory of the University Hospital “Città della Salute e della Scienza di Torino” for the diagnosis of HCMV infection or the monitoring of viremia in immunocompromised/transplanted patients. All samples were collected in K2/EDTA Vacutainer (Becton, Dickinson and Company, Franklin Lakes, USA). Patients’ epidemiological features and involved clinical areas are summarized in Table 1.

**TABLE 1:** Details on the patients’ epidemiology and involved medical areas^*a*^

Patients		Age	
		Mean	Median
Male	183	52	59
Female	140	48	54

Medical area	Patients	Samples
Transplantation unit	119	292
General medicine	116	206
Surgery	38	91
Pediatric general medicine	31	101
ICU/ED	11	32
Pediatric transplant unit	8	33
Total	323	755

^*a*^ ICU, intensive care unit and ED, emergency department.

### qPCR chemistry

Our laboratory SoC procedure, used as a reference in the present study, consisted of two steps: an initial extraction of HCMV DNA from whole blood samples with DSP Mini Kit on the QIAsymphony SP/AS platform (Qiagen, Hilden, Germany) and subsequent amplification with the CMV ELITe MGB qPCR kit (ELITechGroup, Turin, Italy) on the ABI 7500 Fast Dx instrument (Applied Biosystems, Waltham, USA). For each run, a negative template control (NTC control), a positive control, and four defined concentration standards (10^2^, 10^3^, 10^4^, and 10^5^ copies/reaction) were added to validate the qPCR protocol and construct the calibration curve. Once obtained, the results were converted to International Units per milliliter (IU/mL) with the Real Time System Analysis Software V. 2.0 (release 1.9991) (ELITechGroup, Turin, Italy). The evaluated viral target was the exon 4 region of the CMV major immediate early antigen gene (HCMVUL123) with an amplicon size of 79 base pair (bp).

### TMA chemistry

A total of 500 µL of whole blood samples were diluted in a prefilled secondary tube containing 1,425 µL of Aptima Whole Blood Diluent (Hologic Inc., Marlborough, USA). Then, each sample was loaded onto the Panther system automated platform (Hologic Inc., Marlborough, USA) and tested with the Aptima CMV Quant assay kit (Hologic Inc., Marlborough, USA). The assay consists of three main steps: target capture, target amplification using TMA, and detection of amplification products. Finally, the results were converted from copies/mL to IU/mL using a conversion factor equation embedded in the Panther software. For this kit, the viral region tested was the unique long 56 region of the HCMV genome with an amplicon size of 108 bp.

Both chemistries (qPCR and TMA) have been standardized to the WHO 1st International Standard for Human Cytomegalovirus (NIBSC code: 09/162, UK).

### Clinical agreement

Whole blood samples were tested simultaneously with qPCR and TMA chemistries in order to evaluate positive percent agreement (PPA), negative percent agreement (NPA), and quantification agreement. Data obtained were grouped into three subsets: not detected (HCMV viremia not observed), detected not quantifiable (HCMV viremia observed below the limit of quantification), and detected and quantifiable (HCMV viremia within the quantifiable range 2.24–7.0 Log_10_ IU/mL). Moreover, 12 selected patients were monitored for HCMV viremia up to 3 months of hospitalization; the inclusion criteria for patients’ selection were (i) solid organ transplantation, (ii) no less than four quantifications for at least 14 days, and (iii) HCMV viremia detectable in the first blood sampling with at least one of the two tested methods.

### Statistical analysis

Data were analyzed as Log_10_-transformed values. Only samples in which both assays provided quantitative results were considered in the correlation analysis. Assay results were plotted against each other by using linear regression analysis and Bland-Altman plot. The correlation coefficient was calculated using Pearson’s correlation. All analyses were performed using MedCalc Statistical Software version 20.305 (MedCalc Software Ltd, Ostend, Belgium).

## RESULTS

TMA and qPCR performance data showed 99.27% agreement for quantified detected samples and 89.39% agreement for those negative between the two tested methods (Table 2). Cohen’s kappa coefficient was excellent (*K*-value: 0.87).

**TABLE 2:** Clinical qualitative agreement between TMA and qPCR assays

		TMA				Agreement (%)
		Not detected	Detected not quantifiable	Detected and quantifiable	Total	
qPCR	Not detected	337	34	6	377	89.39
	Detected not quantifiable	9	23	71	103	22.33
	Detected and quantifiable	0	2	273	275	99.27
	Total	346	59	350	755	

**Fig 1:**
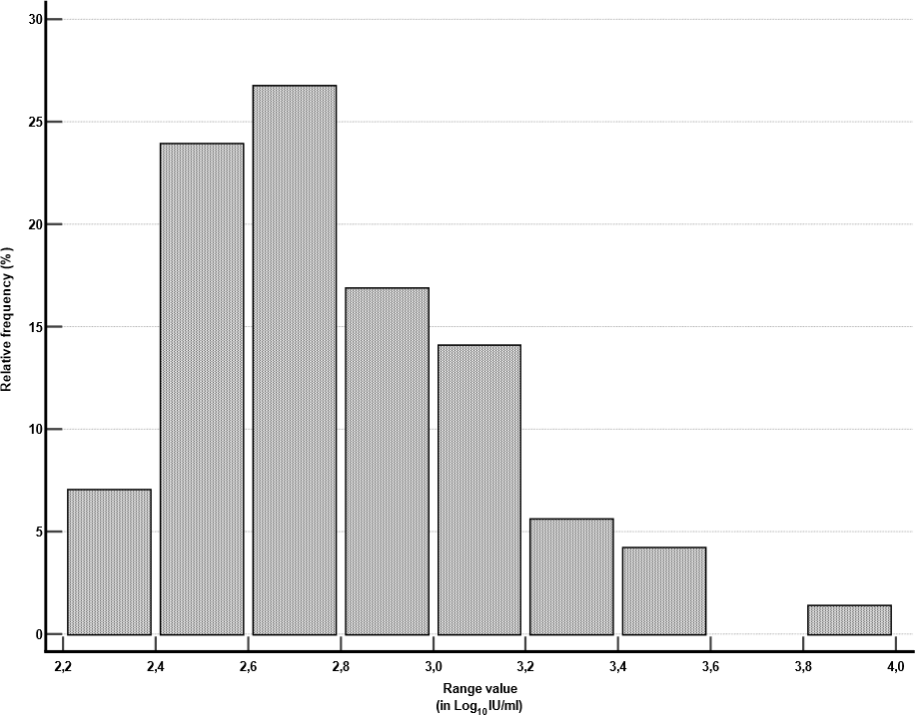
Distribution of Log_10_ IU/mL values with TMA assay in qPCR detected samples.

Compared to conventional qPCR, the TMA assay resulted in a higher number of detected or quantified samples (10.61%): five samples quantified with the TMA chemistry had an average value between 2.27 and 2.84 Log_10_ IU/mL, while one sample had a higher value of 3.92 Log_10_ IU/mL. When repeated, the result was confirmed. Furthermore, for samples quantified with the TMA assay and detected by qPCR, values ranged from 2.0 to 4.0 Log_10_ IU/mL, with the highest distribution between 2.4 and 2.8 Log_10_ IU/mL (36/71, 50.70%) (Fig. 1).

Differently, nine samples tested positive by qPCR but were not detected by TMA, while two were quantified only by qPCR assay: the mean value was 2.65 ± 0.14 Log_10_ IU/mL (Table 2). A total of 273 samples had results within the quantifiable range for both assays and were used for correlation analysis.

**Fig 2:**
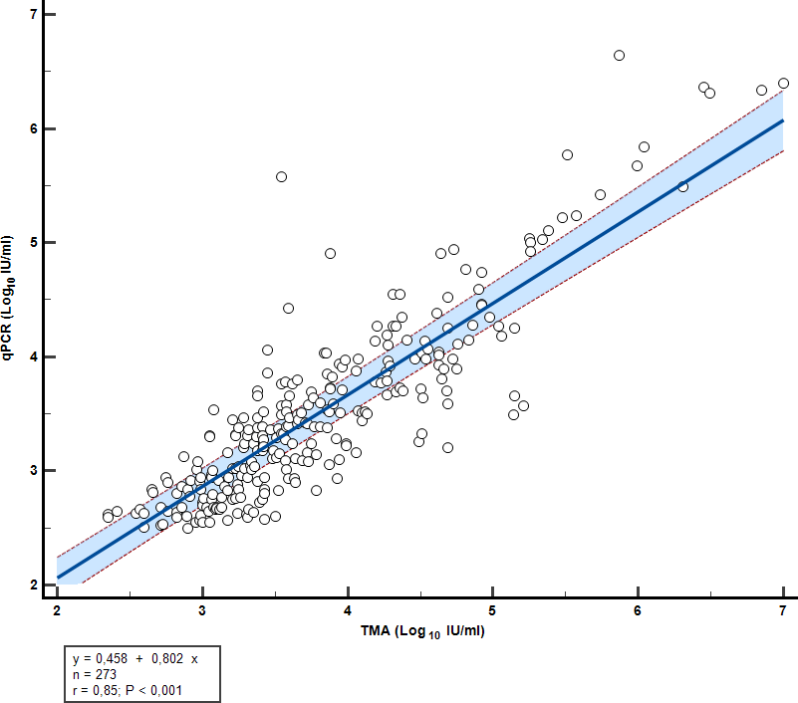
Log_10_-transformed quantitative viral load agreement for TMA and qPCR chemistry: linear regression.

Deming regression analysis showed a significant correlation between the two chemistries: the linear regression line was calculated as follows: *Y* = 0.4581 + 0.8024*x* with an *R*^2^ = 0.7142 (Fig. 2). The Pearson correlation coefficient was 0.8796 (95% CI: 0.8496–0.9039) (*P*-value < 0.0001).

**Fig 3:**
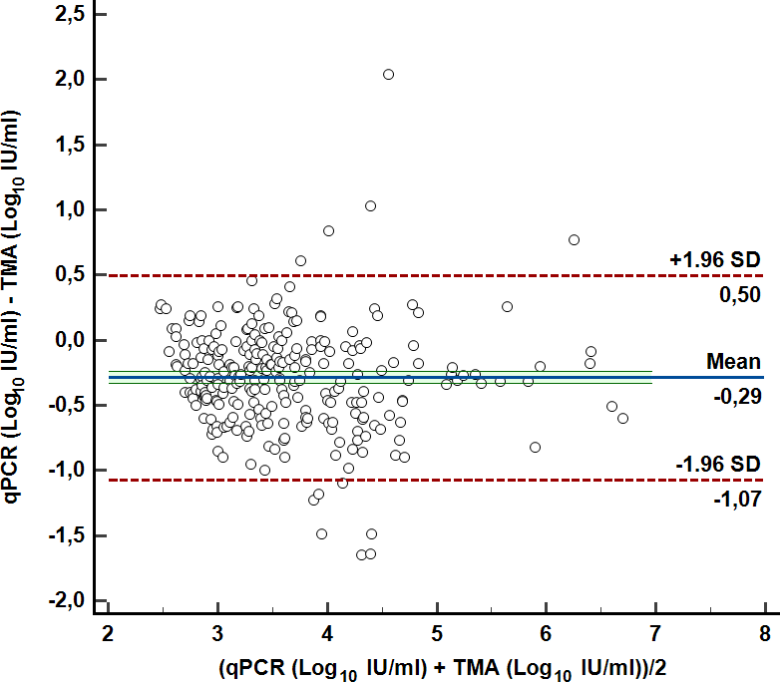
Log_10_-transformed quantitative viral load agreement for TMA and qPCR chemistry: Bland-Altman plot.

The mean viral load (±SD) from the TMA assay was 3.79 ± 0.83 Log_10_ IU/mL, while that obtained by qPCR was 3.50 ± 0.80 Log_10_ IU/mL. Bland-Altman analysis (Fig. 3) yielded a mean bias of −0.29 Log_10_ IU/mL (SD ± 0.40 Log_10_ IU/mL). The lower and upper limits of agreement were −1.07 (95% CI: −1.15 to −0.99) and 0.50 (95% CI: 0.42–0.58) respectively.

**Fig 4:**
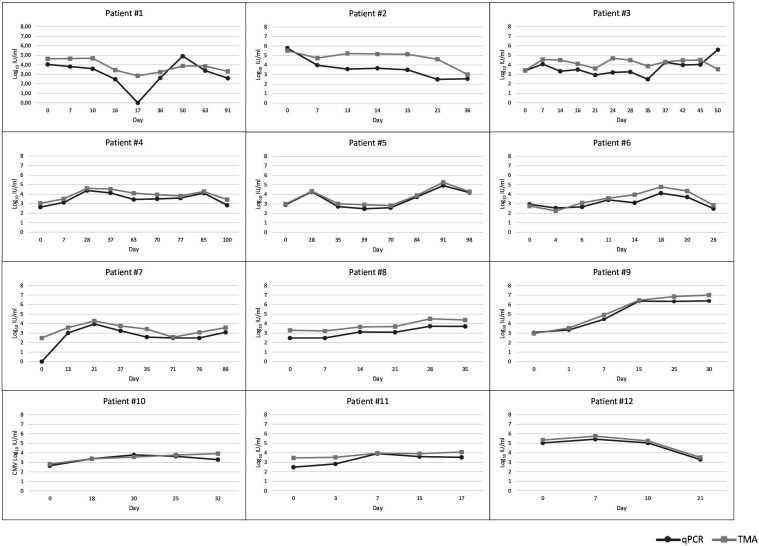
Trend of HCMV viremia with qPCR and TMA assays in transplanted patients.

According to the abovementioned criteria, 12 transplant patients were monitored for a period ranging from 17 to 100 days, depending on the hospital stay (Fig. 4). All patients tested positive for both methods at time 0, except one (Fig. 4, patient #7), whose initial viremia was detectable only by the TMA assay. Overall, in the analyzed samples from these patients, the mean difference between the qPCR and TMA chemistries was −0.59 ± 0.63 Log_10_ IU/mL. The closest agreement resulted for patients #5, #9, #10, and #12 (mean difference −0.23 ± 0.06 Log_10_ IU/mL). Interestingly, patient #1 resulted steadily positive for up to 3 months of monitoring with the TMA assay, while qPCR tested negative on day 17. However, the previous day both tests resulted positive (2.48 or less and 3.45 Log_10_ IU/mL, respectively, for qPCR and TMA chemistry). When retested, the trend of HCMV viremia was confirmed for the samples collected on days 16 and 17 with both assays.

The most evident difference between tested assays resulted in patient #2 with a mean value of −1.12 ± 0.84 Log_10_ IU/mL.

## DISCUSSION

Early detection of HCMV viremia is essential to identify progressing infection and high-risk patients who require antiviral therapy. Particularly, the effectiveness of a preemptive therapy relies on accurate laboratory tests to monitor HCMV infection (27). The specimens currently recommended as the gold standard for monitoring the presence of HCMV DNA are whole blood and plasma. In the first matrix, HCMV is mainly located in monocytes, while in plasma it can be found in the form of viral fragments released by infected cells (21). To date, there is no consensus on the optimal blood compartment for routine molecular testing of HCMV DNA and on the stability of the viral genome in both whole blood and plasma (28, 29). However, the higher sensitivity of whole blood and its greater yield of HCMV DNA make it an optimal sample for monitoring viral load in immunocompromised patients (30). Furthermore, in order to improve interlaboratory agreement on the quantification of HCMV DNA, also according to the first WHO International Standard established in October 2010, different diagnostic workflows (extraction and amplification) were evaluated to define an appropriate conversion factor from genome/mL to IU/mL in both whole blood and plasma in a multicenter study (31, 32). Also, due to this standardization, a further multicentric study has allowed a better understanding of viral kinetics and threshold values for predicting HCMV disease. In fact, although peak viral load was reached simultaneously in the two blood compartments, HCMV viremia in whole blood was consistently higher (approximately 1 Log_10_ IU/mL) than in plasma from the onset of infection until peak viral load. Moreover, viremia in whole blood decreased more rapidly than in plasma, thus avoiding unnecessary prolonged treatment and associated drug toxicity and may result in cost savings (33).

Several qPCR diagnostics assays have been developed and are currently available for the identification and quantification of HCMV in plasma and whole blood; in addition to these, the Aptima CMV Quant Assay, a new TMA-based assay, has also recently been introduced.

Unlike qPCR, TMA is an isothermal molecular method based on microbial RNA via a reverse transcription step followed by amplification and is capable of producing 100–1,000 copies per cycle (compared to the two copies per cycle of qPCR).

Although the correlation between qPCR and TMA chemistries for HCMV detection has been evaluated on plasma and other sample types with excellent concordance (34, 35), EDTA whole blood as a clinical sample has not yet been investigated.

In this study, we evaluated the performance of the TMA assay (Aptima CMV Quant Assay) and compared the data with our qPCR SoC (CMV ELITe MGB Kit). The clinical qualitative agreement on whole blood between the two chemistries was excellent (99.27% and 89.39% for the PPA and NPA, respectively). Moreover, the TMA assay quantifies on average 0.29 Log_10_ IU/mL higher than the qPCR assay, as shown in the Bland-Altman plot (Fig. 3). Since the mean difference was less than the cut-off (0.5 Log_10_ IU/mL) for considering load changes to be biologically important (36), our results suggest no clinical implication if the diagnostic platform is changed. Indeed, data from plasma samples showed a mean difference varying between –0.20 and 0.58 Log_10_ IU/mL, according to the TMA assay compared to different qPCR platforms (34, 35).

In our work, the TMA assay was more sensitive, resulting in a higher number of samples with a defined viral load (Table 2). This is probably due to the differences between the two methods in terms of the limit of quantification (LoQ). On whole blood, it was 2.24 Log_10_ IU/mL and 2.48 Log_10_ IU/mL for TMA and qPCR, respectively. Several discrepant samples were in the range of 2.2–2.8 Log_10_ IU/mL, thus close to the lower LoQ of the qPCR assay (Fig. 1). Interestingly, the TMA assay required a higher extraction volume (700 µL) that had to be diluted 2.85-fold with the appropriate preparation buffer, whereas for qPCR, the starting volume was 200 µL of whole blood directly from the primary tube without the need for dilutions. Moreover, the TMA assay was evaluated with a fully automated sample-to-result platform, while two different platforms for HCMV DNA extraction and amplification, respectively, were used jointly for our SoC qPCR. All the abovementioned aspects, together with gene target and amplicon size (37), must be taken into account to explain the differences between the two tested methods.

To our knowledge, this is the first time that the performance of TMA chemistry has been evaluated to monitor viremia in transplant patients; 12 individuals were selected and tested for a time span ranging from 3 weeks to more than 3 months with both assays. Our data showed a good correlation between the two methods, with TMA values on average higher than qPCR (Fig. 4), in agreement with the overall results. The largest discrepancy was observed in patient #1, where qPCR was suddenly undetected, while TMA assay showed a linear trend in the detection of HCMV viremia.

The excellent results of the TMA assay in the present study, along with those of other studies, provide the opportunity for further studies to better understand the impact of this promising technology, especially in transplant patients, clinical management, and the decision to begin preemptive therapy or antiviral prophylaxis. In addition to clinical and analytical performance, the TMA assay showed the advantage of being carried out on a fully automated system with random and continuous access, thus eliminating the need for sample batching and leading to faster turnaround time. The availability of an automated platform with minimal hands-on time also enables timely diagnosis, prognosis, and monitoring of HCMV-infected patients.

Finally, this is the first TMA kit with FDA approval on plasma and CE-IVD on both whole blood and plasma samples, so clinical laboratories can easily incorporate this assay into their routine molecular workflows. However, due to its recent introduction for the diagnosis of HCMV, no comparison data between plasma and whole blood are available to assess the most suitable matrix with TMA chemistry. Limitations of our study include the small number of patients enrolled for the follow-up and the lack of correlation with antiviral therapy, selection of drug-resistant viral strains, and patient clinical outcome. In addition, there were no specifics available about the HCMV genotypes in the positive samples tested. Laboratories evaluating a change to a different diagnostic system should considered all previously mentioned aspects by performing side-to-side testing using both current and new assays to understand the most suitable diagnostic workflow and clinical matrix.
